# Corticosteroids and RCTs against the supposed undervaluation of real data evidence

**DOI:** 10.1186/s13054-021-03721-3

**Published:** 2021-08-18

**Authors:** Alejandro Rodríguez, Gerard Moreno, Maria Bodi, Josep Gomez, Ignacio Martín-Loeches

**Affiliations:** 1 ICU Hospital Universitario Joan XXIII/IISPV/URV, CIBERes, Tarragona, Spain; 2 Tarragona Health Data Research Working Group (THeDaR), ICU Hospital Universitario Joan XXIII, Tarragona, Spain; 3 Hospital Clinic, IDIBAPS, Universidad de Barcelona, CIBERes, Barcelona, Spain; 4 Multidisciplinary Intensive Care Research Organization (MICRO), Department of Intensive Care Medicine, St. James’s Hospital, Dublin 8, Dublin, Ireland

## Dear Editor

The alleged supremacy of the Randomized control trials (RCTs) is based on the possibility of causal inference. But, is this true?

In order to define what we mean by a causal effect, for each subject we assume the existence of the potential outcomes *Y*^*α* = 0^ and *Y*^*α* = 1^ corresponding to what value the outcome would take if we do not apply the intervention (*α* = 0) or we apply the intervention (*α* = 1), respectively. For an individual, it is assumed that the intervention has a causal effect whenever *Y*^*α* = 0^ different to *Y*^*α* = 1^, that is, the outcome would take a different value depending on whether the individual is given the intervention or not. To calculate the causal effect of the intervention we would need to somehow obtain or discover the values *Y*^*α* = 0^ and *Y*^*α* = 1^. Consider that “*A*” denote a random variable indicating whether an individual receives the intervention (*A* = 1) or not (*A* = 0), and Y a random variable for the observed outcome. If a particular individual received the intervention, the observed value is *Y* = *Y*^*α* = 1^, but for the potential outcome value *Y*^*α* = 0^ is unknown. The unobserved outcome is called the “counterfactual” outcome and a causal inference is not possible to obtain [1].

According to the RECOVERY trial results, an adaptive RCT [2], during the second pandemic wave, corticosteroid use was generalized in all critical COVID-19 patients. However, the role of corticosteroids in the treatment of COVID-19 remains controversial. A recent study [3] comparing first versus second wave reported that, despite of the systematic and early administration of glucocorticoids in the second wave, the ICU mortality (50% vs. 52%, *p* = 0.96) and of ICU length of stay did not differ between the two waves.

Late complications, as well as medium-term evolution, were not evaluated in the RECOVERY study [2]. A recent research letter [4] found that corticosteroid use, and tocilizumab treatment were associated with ventilator-associated pneumonia (VAP).

Should physicians continue to ignore the results of well-adjusted observational studies in favor of the results of an adaptive RCT with many inconsistencies? We are convinced that steroid treatment can be effective but there are different patients’ phenotypes and their use should be re-evaluated [5].

Possibly, the future of research and clinical practice is not conceived as a confrontation between RCT and observational studies, but rather as the sum of knowledge between RCTs and its clinical application.

## Data Availability

Not applicable.
